# Accuracy of warm ischemia time measurement using a surgical intelligence software in partial nephrectomies: A validation study

**DOI:** 10.1002/bco2.452

**Published:** 2024-10-26

**Authors:** Archan Khandekar, Joao G. Porto, Jean C. Daher, Pedro F. S. Freitas, Dotan Asselman, Maritza M. Suarez, Mark L. Gonzalgo, Dipen J. Parekh, Sanoj Punnen

**Affiliations:** ^1^ Desai Sethi Urology Institute, Miller School of Medicine University of Miami Miami Florida USA; ^2^ Theator Inc. Palo Alto California USA; ^3^ Department of Medicine University of Miami Miller School of Medicine Miami Florida USA

**Keywords:** artificial intelligence, computer vision, partial nephrectomy, renal tumour, warm ischemia time

## Abstract

**Objectives:**

The objectives of this study are to compare the accuracy of warm ischemia times (WITs) derived by a surgical artificial intelligence (AI) software to those documented in surgeon operative reports during partial nephrectomy procedures and to assess the potential of this technology in evaluating postoperative renal function.

**Patients and methods:**

A surgical AI software (Theator Inc., Palo Alto, CA) was used to capture and analyse videos of partial nephrectomies performed between October 2023 and April 2024. The platform utilized computer vision algorithms to detect clamp placement and removal, enabling precise WIT measurement. Expert‐reviewed surgical videos served as the ground truth. Platform‐derived WITs were compared to those in surgeon operative reports using paired‐sample *t*‐tests. Additionally, we analysed the correlation between platform‐derived WITs and postoperative creatinine levels extracted from electronic health records (EHRs) integrated via health level seven (HL7) messaging protocols.

**Results:**

Of 64 eligible cases, 61 were included in the final analysis. Platform‐derived WITs were within 1 min of the ground truth in all procedures, within 30 s in 97%, and within 10 s in over 80%. The mean difference between platform‐derived WITs and ground truth was 8.3 s, significantly lower than the 2.45 min difference for operative reports (*p* < 0.001). No significant correlation was found between platform‐derived WIT and postoperative creatinine changes, aligning with the view that WIT may not independently determine postoperative renal function. Although not the primary goal of this study, significant correlations were observed between WIT, tumour size and RENAL score.

**Conclusion:**

This study demonstrates the high accuracy of a surgical intelligence platform in measuring WIT during partial nephrectomies. The findings support the use of AI‐based surgical time measurement for precise intraoperative documentation and highlight the potential of integrating these data with EHRs to advance research on surgical outcomes.

## 1 INTRODUCTION

Methods of time measurement during surgery have evolved significantly over the years. Historically, surgical teams estimated times and recorded them manually, relying on wall clocks, wristwatches or even sand timers. Electronic timers and digital clocks later led to improved accuracy and reliability. Today, information about surgical times comes almost exclusively from postoperative narrative reports, which are rarely comprehensive or accurate.^1^, ^2^

Precise documentation of surgical times can be critical, particularly when there is reason to believe that the durations of specific steps or events could affect postoperative outcomes. A notable example is warm ischemia time (WIT) in partial nephrectomy. Research examining the effects of WIT on postoperative renal function is inconclusive, with some studies suggesting that prolonged ischemia can lead to substantial damage and others attaching greater importance to patient characteristics and other surgical factors.^3^, ^4^, ^5^ This debate is reflected in clinical practice, leading to substantial variability in surgical guidelines, technique and decision‐making.^6^

Studies showing discrepancies between reported and actual clamp times indicate that imprecise WIT measurements could contribute to the inconsistent literature on the relationship between WIT and renal function. Indeed, if we are unable to measure and document intraoperative activity accurately, we cannot expect to accurately assess its impact.

The emerging field of surgical artificial intelligence (AI) addresses this gap.^7^ Installed in operating rooms and implemented in daily workflows, surgical intelligence platforms use AI to enable routine, automated capture, storage and analysis of surgical video. Alongside other critical intraoperative data, these platforms document the times and durations of key surgical steps and events. With all relevant procedures performed in a department routinely recorded and automatically analysed according to well‐defined visual criteria, the method is both highly scalable and substantially less susceptible to the errors and biases associated with traditional data collection. Integrating Theator with electronic health records (EHRs) using health level seven (HL7) messaging protocols allows seamless data scaling to other datasets automatically, minimizing human intervention including and not limited to comparator data entry. Moreover, surgical intelligence platforms integrate AI‐derived intraoperative data with EHR data regarding postoperative outcomes, enabling accurate assessment of ties between them.

The platform is designed to integrate and analyse surgical videos from laparoscopic, robotic and endoscopic procedures. It evaluates multiple parameters, making this use case just one of many potential applications. As a result, determining the cost specifically for this use case is challenging.

In the context of partial nephrectomy, computer vision algorithms that automatically detect clamp placement and removal allow surgical intelligence platforms to quantify WIT. In the current study, we compared the accuracy of WITs derived by a surgical AI platform to that of WITs documented in surgeon operative reports, with expert review of surgical videos serving as the ground truth. We aimed to demonstrate the potential utility of this technology in examining the effects of WIT on renal functioning, while providing primary support for AI‐based surgical time measurement in general.

## 2 PATIENTS (OR MATERIALS) AND METHODS

The study was conducted at the Desai Sethi Urology Institute at University of Miami where an installed surgical AI platform (Theator Inc., Palo Alto, CA) is used routinely to capture, analyse and securely store procedure videos. Theator's platform monitors all cases performed in the operating room across various specialties, enabling comprehensive analysis and recognition of different surgical modalities. This capability is crucial for ensuring the accurate and consistent video documentation and analysis of surgical procedures, which can be integrated with patient outcomes data. The study included videos of all partial nephrectomies conducted between October 2023 and April 2024 to treat renal tumours, excluding those with more than one tumour and those with two or more completely discrete ischemia events.

### 2.1 Computer vision application and EHR integration

The employed surgical AI platform incorporates validated computer vision algorithms that accurately break down surgical videos into procedure‐specific steps and identify intraoperative actions and events in a range of procedure types among them partial nephrectomy.^8^ An integrated algorithm that blurs extracavitary frames maintains patient and surgeon confidentiality, in accordance with published standards for surgical video de‐identification.^9^, ^10^

In the context of partial nephrectomy, the platform incorporates state‐of‐the‐art computer vision models that have previously been trained to detect visual cues indicating the application and the removal of arterial bulldog clamps, marking the beginning and end of ischemia, and enabling accurate calculation of WIT.

In brief, there are two main components: (1) a base model that processes the raw surgical video frames and (2) an event detection model that identifies temporal segments in which surgical actions occur. For the base model, a vision transformer network (VTN) inputs the video and provides a numerical representation, by learning spatial and temporal information presented in the video.^7^, ^11^ The event detection model uses a temporal action localization approach on top of the VTN representation. Training both models in an end‐to‐end manner enables the achievement of a robust system that can identify both the application and removal of arterial bulldogs and the temporal localization. Together, these produce an automatic and accurate inference of WIT. Algorithm accuracy is maintained across varying conditions within the operating theatre, adjusting for factors such as lighting variations and movement blur.

#### 2.1.1 EHR integration

The Theator AI platform integrates a robust interface with the Epic EHR system, utilizing HL7 messaging protocols. This integration streamlines the transfer of intraoperative data, allowing for automatic scalability to other datasets within or across healthcare systems without the need for manual intervention. This scalability is imperative for extending research findings to broader populations and enhancing the generalizability of the results.

### 2.2 Measures and statistical analyses

Study measures included WIT and the durations of the tumour resection and resection site closure steps, as identified automatically by the surgical AI platform, and the following measures extracted from the EHR: WIT as reported in the surgeon operative report, patient age and BMI, RENAL score, tumour size and postoperative change in creatinine level.^12^

We defined the ground truth WITs for all included cases based on the agreement of three senior surgeons, who reviewed the intraoperative videos. To directly compare the accuracy levels of WITs derived from the platform to those derived from the operative reports using a paired‐sample *t*‐test. We then calculated the mean difference between each of these variables and the ground truth WITs and conducted a paired‐sample *t*‐test to compare the differences. Finally, we assessed partial correlations between the following variables: platform‐derived WIT, platform‐derived tumour resection and resection site closure durations, RENAL score, tumour size and change in creatinine.

## 3 RESULTS

Of the 64 cases that met the inclusion criteria, 61 were included in the final dataset. Three cases were excluded: one due to the presence of multiple tumours, another due to multiple ischemia events and the third due to a WIT that was more than four standard deviations higher than the mean. Of the procedures documented, 43% were performed on males. Platform‐derived measures and demographic and clinical characteristics of the sample are presented in Table 1.

**TABLE 1:** Means and standard deviations of study variables, including platform‐derived warm ischemia time (WIT), tumour resection and resection site closure durations, and demographic and clinical characteristics.

	Median	IQR
Age (years)	64	20
BMI (kg/m^2^)	28.9	6.50
RENAL score (*N* = 60)	7.00	3.00
Tumour size (cm)	3.50	1.90
Operative time (hours)	1.97	1.13
WIT (minutes)	21.9	8.47
Tumour resection duration (minutes; *N* = 60)	6.15	4.71
Resection site closure duration (minutes; *N* = 59)	14.8	7.10
Postoperative change in creatinine (mg/dL)	0.160	0.200

*Note*: *N* = 61, unless otherwise specified.

WITs identified by the platform were found to be within 1 min of the WITs identified and agreed upon by three senior surgeons (ground truth) in 100% of procedures, validating the accuracy of the platform. Notably, platform‐derived durations were within 30 s of ground truth durations in 97% of procedures and within 10 s in over 80%.

**FIGURE 1:**
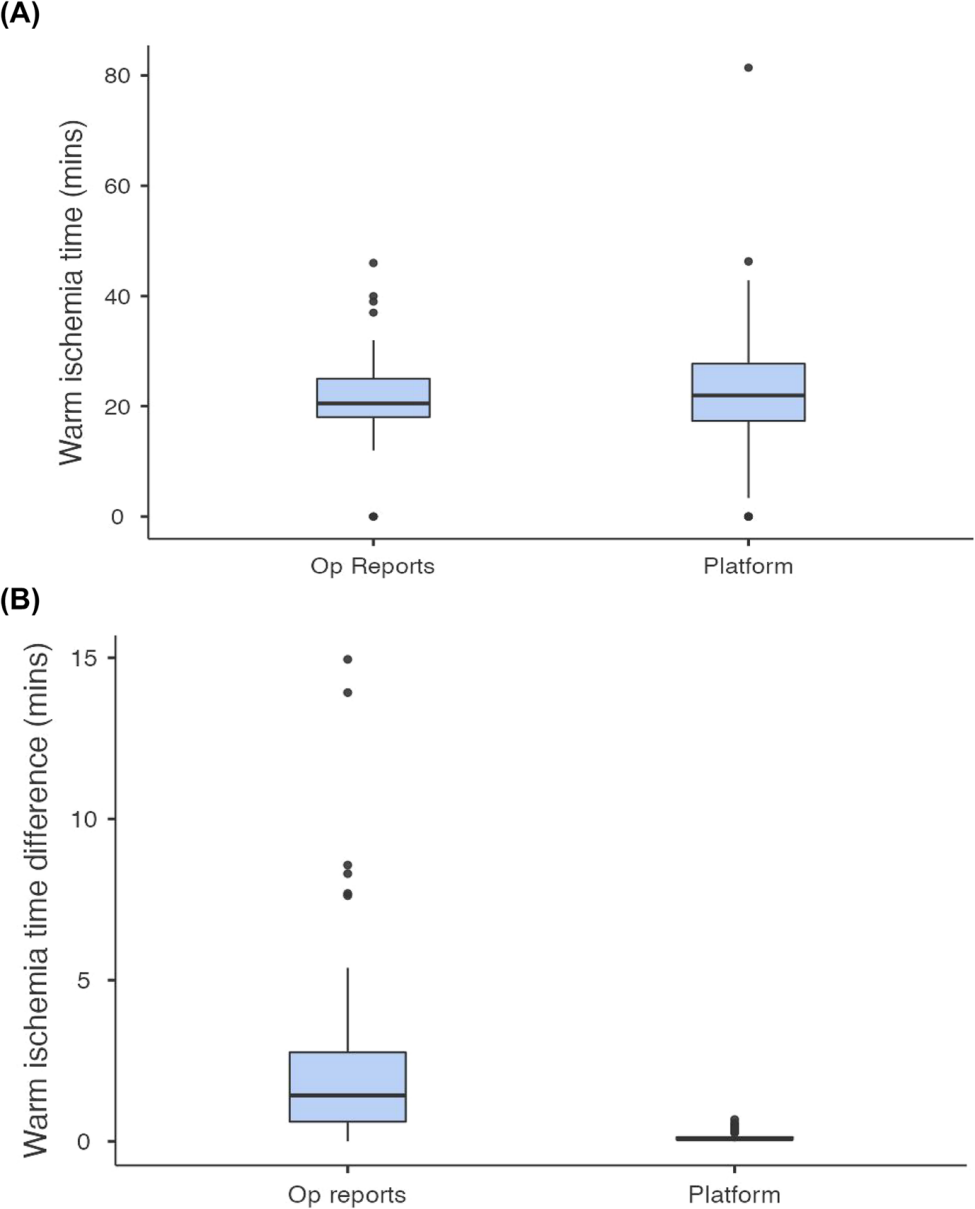
Comparisons between (A) the warm ischemia times (WITs) determined by the platform and written in the operative (Op) reports and (B) the differences from the ground truth WITs, in the platform and the Op reports. Both analyses yielded significant differences, indicating that the WITs in the Op reports were less accurate than the platform‐derived WITs, as compared to the video‐derived ground truth.

Comparing the platform‐derived WITs to the WITs reported in the surgeon operative reports showed a mean difference of 2.39 min (SD = 3.1 min). A paired‐sample *t*‐test showed that this difference was statistically significant, t(59) = 2.24, *p* = 0.029 (Figure 1A). The mean difference between platform‐derived WITs and ground truth WITs was 8.3 s (SD = 9.2 s), while the mean difference between operative report‐derived and ground truth WITs was 2.45 min (SD = 3 min). Another paired‐sample *t*‐test showed that platform‐derived WITs were significantly more similar to the ground truth WITs than were the operative report‐derived WITs, t(59) = 5.90, *p* < 0.001 (Figure 1B).

As shown in Table 2, WIT was closely correlated with both tumour resection and resection site closure durations, but the latter two variables were not correlated with one another. WIT was also correlated with both tumour size and RENAL score. Change in creatinine was not correlated with WIT, tumour resection duration or resection site closure duration. This was also the case when we ran the analysis again with RENAL score and tumour size controlled.

**TABLE 2:** Results of partial correlation analyses between study variables.

		Warm ischemia time	Tumour resection duration	Resection site closure duration	RENAL score	Tumour size
Warm ischemia time	Pearson's *r*	–	–	–	–	–
	*p*‐Value	–	–	–	–	–
Tumour resection duration	Pearson's *r*	0.548***	–	–	–	–
	*p*‐Value	<0.001	–	–	–	–
Resection site closure duration	Pearson's *r*	0.737***	0.167	–	–	–
	*p*‐Value	<0.001	0.206	–	–	–
RENAL score	Pearson's *r*	0.433***	0.343**	0.376**	–	–
	*p*‐Value	<0.001	0.007	0.003	–	–
Tumour size	Pearson's *r*	0.450***	0.530***	0.189	0.427***	–
	*p*‐Value	<0.001	<0.001	0.152	<0.001	–
Creatinine change	Pearson's *r*	0.19	0.24	0.102	0.257*	0.370**
	*p*‐Value	0.142	0.065	0.442	0.046	0.003

* *p* < 0.05.

** *p* < 0.01.

*** *p* < 0.001.

## 4 DISCUSSION

The current findings demonstrate the accuracy of a surgical AI platform in documenting WIT during partial nephrectomy procedures. In reference to the video‐based, expert‐defined ground truth, platform‐derived WITs showed a discrepancy of only 8 s, as compared to almost 2.5 min for surgeon operative reports. All platform‐derived WITs fell within 1 min of ground truth durations. This ability to automatically and routinely provide precise WIT measurements highlights the potential of surgical intelligence to transform the way we collect and analyse surgical data.

The application of computer vision technologies has opened new possibilities for precise and objective documentation of intraoperative activity. By leveraging these technologies, researchers can now capture and analyse surgical video data in real‐time, automatically detecting and recording critical events. For example, in laparoscopic cholecystectomy, computer vision models have been developed to automatically locate and document the critical view of safety, a key operative step that ensures proper identification of hepatocystic anatomy to prevent bile duct injury.^13^ This approach eliminates the potential for human error and bias, providing a more faithful representation of the surgical reality across various procedures.

Specifically in the context of WIT and renal function, accurate documentation of surgical times could play a role in settling a longstanding debate, as the intricate interplay between these variables remains a critical focal point within the domain of urological surgery.^14^, ^15^ In the early 2000s, researchers began to explore the link between WIT and postoperative kidney function. Thompson and colleagues investigated the factors affecting kidney function after partial nephrectomy in a diverse group of patients with tumours in solitary kidneys, comparing the effects of cold and warm ischemia during the surgery.^4^ Initially, the results confirmed that ischemia time was a significant factor in determining postoperative kidney function. However, in a subsequent study by the same group, when changes in the volume of functioning kidney tissue were accounted for, ischemia time lost its significance, and the amount of kidney tissue saved became the most crucial factor.^16^

The CLOCK trial, a randomized controlled trial involving 324 patients, revealed that ischemia times exceeding 10 min had an impact on estimated glomerular filtration rate.^17^ However research by Parekh et al. through systematic renal biopsies done before and after the partial nephrectomies questioned the threshold for ischemia time as they found no link to kidney injury or progression of chronic kidney disease.^18^ Instead, factors such as existing conditions like hypertension and diabetes were identified as better predictors of deterioration.^19^ The inconclusive literature highlights the need for more precise time measurement in partial nephrectomy that can be scaled to give ‘big data’ arguments on both sides.

In the present study, beyond demonstrating accurate AI‐based measurement of WIT, we conducted a preliminary analysis of the relationship between platform‐derived WIT and EHR‐derived postoperative change in creatinine. The lack of correlation appears to align with the view that WIT is not an independent determinant of postoperative renal function. While we note that early creatinine levels are not an ideal or sufficient indication of renal function after partial nephrectomy, there has been work to show that even the immediate postoperative creatinine, at postoperative day 1, is a strong predictor of eventual chronic kidney disease.^20^ We included this analysis to demonstrate the potential of surgical intelligence platforms to integrate intraoperative and EHR data in an accurate, scalable manner. By leveraging HL7 standards, a set of international standards for the exchange, integration, sharing and retrieval of electronic health information, this data collection process can be extended across multiple healthcare systems and extrapolated to larger populations.^21^

Our findings also revealed significant correlations between WIT, tumour size and RENAL score, indicating that more complex partial nephrectomies were associated with longer WITs. In line with prior research, this finding reveals the challenge of isolating the contributions of interrelated variables in determining renal outcomes.^9^ Namely, if larger tumours are associated with longer WITs, and both are correlated with poorer outcomes, it can be difficult to tell which factor plays the more dominant role. To address this issue requires large, diverse study samples, in which complexity measures like tumour size can be stratified and the separate and interactive effects of multiple variables can be examined. This highlights an additional advantage of surgical intelligence platforms, as routine, automated capture and analysis enables larger, more diverse datasets than could previously be collected.

The current findings' generalizability is limited by the small sample size and the presence of confounding factors. However, the study serves as an initial proof‐of‐concept for using image capturing and application of a machine learning model to assess surgical times and their effects on postoperative outcomes. The innovative methods described here can be applied in future research, using larger, more diverse patient populations and longer follow‐up periods.

## 5 CONCLUSIONS

This study validates the accuracy of a surgical AI platform in measuring WIT during partial nephrectomies and demonstrates the feasibility of integrating and scaling these data using HL7 standards. As healthcare advances towards an era of big data, such platforms and data integration methods will be essential in understanding the complex factors affecting surgical outcomes and driving progress in the field.

## AUTHOR CONTRIBUTIONS

Archan Khandekar was involved in all aspects of the research, including conceptualization, design, data collection, analysis and manuscript preparation. Joao G. Porto contributed to the conceptualization and design of the study. Jean C. Daher played a key role in data collection. Pedro F. S. Freitas and Dotan Asselman were responsible for data analysis and interpretation. Maritza M. Suarez and Mark L. Gonzalgo provided clinical expertise and critically reviewed the data. Dipen J. Parekh and Sanoj Punnen supervised the project and contributed to the final manuscript preparation and review.

## CONFLICT OF INTEREST STATEMENT

None.
